# Artificial Intelligence in Rheumatology: A Comprehensive Bibliometric Analysis and Current Scientific Mapping Research

**DOI:** 10.31138/mjr.211025.err

**Published:** 2026-06-01

**Authors:** Maria Polyzou, Johanna Mucke, Anna Kernder, David Kiefer, Xenofon Baraliakos, Philipp Sewerin

**Affiliations:** 1 Department of Pathophysiology, School of Medicine, National and Kapodistrian University of Athens, Laiko General Hospital, Athens, Greece;; 2 Rheumazentrum Ruhrgebiet, Ruhr-University Bochum, Herne, Germany;; 3 Hiller Research Centre, University Hospital Düsseldorf, Medical Faculty of Heinrich Heine University, Düsseldorf, Germany

**Keywords:** artificial intelligence, machine learning, large language models, rheumatology, rheumatic diseases, rheumatoid arthritis, osteoarthritis, spondyloarthritis, VOS viewer

## Abstract

The bibliometric analysis presented in this article delves into the use of Artificial Intelligence (AI) in Rheumatology, aiming to fill a gap in the existing relevant scientific literature. In the article a holistic comprehensive overview of key trends and research clusters in the field are provided, exploiting a number of widely recognised bibliometric techniques, such as citation analysis, co-authorship analysis, co-occurrence analysis, and bibliographic coupling analysis. Notably, the citation analysis reveals a diverse array of highly cited papers, underscoring the multidimensional nature of research in rheumatology. The co-authorship analysis illuminates complex collaborative networks among countries, with prominent clusters such as the European, USA and the Asian-Pacific clusters, highlighting the dynamic and interconnected nature of international collaborations. The co-occurrence analysis identifies four thematic clusters, emphasising the interconnectedness of rheumatic diseases, prediction methods, artificial intelligence algorithms considerations and patient characteristics. Addressing limitations, including the potential bias introduced by specific keywords and database restrictions, in conclusion, the article provides valuable insights for researchers, paving the way for further refinements in understanding the evolving use of AI in rheumatology.

## INTRODUCTION

The field of rheumatology includes a wide range of medical conditions that affect the quality of life of patients while in several cases leading to mortality. A large number of people do not know about many of the rheumatic diseases, and there is a widespread lack of awareness about the complexity and enormous importance of this field of medicine. The cost of treating people with rheumatic diseases is high for health systems in all countries.^1^

Rheumatology faces a critical shortage of healthcare professionals, exacerbated by an aging patient population and rising costs of care. Developments in digital health technologies (DHT), observed in recent years, offer new opportunities to address new challenges and could contribute to the overall improvement of healthcare in this field.^2^

Recently Artificial Intelligence (AI) and its specific subset techniques such as Machine Learning (ML) and Large Language Models (LLM) have been integrated into the diagnosis and therapy process. The ability of a computer system to perform functions that normally require human intelligence is known as AI. The application of AI is widespread in science and is also used in healthcare. There are potential applications for AI in almost all areas of healthcare, including disease diagnosis, medical image processing, drug development and public health assessment and prediction.^3,4^ The continued development of stochastic technologies has led to significant improvements in AI, most notably the introduction of machine learning and deep learning.

AI and its techniques applications on Rheumatology were becoming increasingly popular, while the adoption of AI techniques by the various rheumatology research groups is increasing.^5,6,7^ This is indirectly determined by the increasing number of publications, in recent years, in specialised rheumatology journals of high impact.^4,8,9^ Despite the recognition of the increasing use of AI and its techniques in the management of rheumatic diseases, the international literature has limited comprehensive bibliometric analyses of the current state of research in AI and rheumatology. We will refer to three characteristic recent publications, which contain bibliometric analyses on the relationship between AI and rheumatic diseases. Liu et al. (2024) in a bibliometric analysis they conducted on the use of Artificial Intelligence in rheumatoid arthritis for the period from 2004 to 2023 outlined the current status and future research trends and directions.^10^

Zhao et al. (2024) also conducted a bibliometric analysis to explore the current research centres and collaboration networks in the application of AI in rheumatic diseases in recent years.^11^ Finally, Polyzou and Baraliakos (2025) assessed some interesting bibliometric indicators concerning the applications of AI in rheumatology contained in the bibliography of the period 2010 to 2024, and attempted to verify the application of Lotka’s law and Bradford’s law for the scientific productivity of the authors in the field of these applications.^12^ Bibliometrics, a quantitative research technique for examining the academic qualities of literature, aids in locating research hotspots and trends in a specific area and forecasting its future prospects.^4^

This study aims to fill a small part of this gap by carrying out an extensive bibliometric analysis, offering an overview of authors, main trends, research groups, and shortcomings in this scientific field. The study is based on a comprehensive sample of articles published by international journals and indexed in the Scopus database.

In a general sense, any bibliometric analysis aims to reveal the structure of a given scientific field, identifying influential works, prolific authors, collaborative networks, and emerging research topics.^13,14^ In this context, the present article aims to organiσe the volume of academic production related to the use of Artificial Intelligence in rheumatology and to facilitate researchers in the process of identifying trends and gaps in the literature. It also provides a roadmap for understanding the key contributions, influencers, and potential areas of influence in the field under study.

In summary, in this article, we aim to objectively assess the impact of research knowledge related to the above keywords on rheumatology topics using databases of published relevant literature, to show the evolution of the number of published articles over time, and to illustrate the collaborations of researchers on related topics. In this context, the article deals with the quantitative analysis of citations, the number of citations, the importance of a research topic, and the research conducted in certain geographical areas. Like any bibliometric analysis, the one conducted in this article can help uncover emerging trends in a journal, article performance, collaboration patterns, and research components, making sense of large volumes of unstructured data. Thus, a well-designed bibliometric study can build a solid foundation for advancing a field in new and meaningful ways: it enables scholars to gain a detailed overview, identify research gaps in a particular area, get new ideas for research, and position these studies in terms of their expected contribution to the field.

The contribution or added value of bibliometric analysis regarding the relationship between AI and rheumatology discussed in this article, briefly concerns: Understanding the research field in question, the quantitative assessment of its development and the depiction of trends in the literature. In addition, recording the changes and dynamics of author interest, as well as the emergence of new topics.

By using appropriate keywords, identifying the main areas on which research focuses and identifying the main applications of AI in rheumatology that receive the most attention.

Identifying the most influential publications and authors, which facilitates the use of basic literature by researchers.

Identifying the most active institutions and the countries where they belong, assisting in their benchmarking efforts.

Identifying journals where the most important research in this field is published, which helps researchers target their submissions and keep them informed of the latest findings.

Identifying networks and collaboration patterns between researchers, institutions and countries, highlighting existing and potential opportunities for future collaborations.

Understanding the connections of research on the use of AI in rheumatology with other fields, such as computer science, medical imaging and genetics.

In summary, the article provides a valuable understanding of the evolution and main features of the field of AI and rheumatology, serving researchers, clinicians, policy makers, and patients.

## ANALYSIS OF THE CONCEPTS AI, ML AND LLMs

Understanding the concepts of AI, ML and LLM is a key prerequisite for understanding the interaction of technology in healthcare and their application in this field. Then, these concepts will then be briefly analyσed and their use in the healthcare field will be described.

In general, AI is related to the development of intelligent systems that seek to imitate human behaviour by performing tasks that typically require human intelligence. Such tasks include speech recognition, natural language processing (NLP), text generation and translation, video, audio, and image creation, etc. In modern times, the use of AI in the healthcare industry is constantly increasing, with applications in medical imaging and diagnosis, disease prediction and treatment, robotic surgery, rehabilitation and physical therapy using wearable devices with AI, personal health monitoring, and more.^16^ Regarding the use of artificial intelligence in rheumatology, it includes many potential applications, such as clinical diagnosis and decision-making, image analysis to aid in diagnosis and clinical assessment, disease course prediction, omic data analysis, medical education, etc.^17^

ML is a specific subset of AI techniques that can automatically learn from the data they are presented with, mostly using ground truth data as training sets (i.e., supervised learning). ML is a critical component of many AI systems. ML algorithms are used to train AI models by providing them with datasets derived from past experience and historical data. The model then learns the underlying patterns in the training data, allowing it to make accurate predictions or decisions on new, unseen data. ML models can adapt and improve their performance with continuous data feed.^20^

The significant progress that has been made in the use of ML is mainly the result of the increase in available data and improvements in algorithms, allowing the identification of complex patterns and correlations within data sets. In the healthcare sector, the significant increase in information about the human body system, due to advances in high-throughput sequencing technologies, electronic health records, and medical imaging, has given impetus to the use of ML in this field.^20,21^

The latest development in the field of AI has been the development of transformer models that have improved the understanding of meaning and relationships between data. The advent of transformers has enabled the wider application of AI LLMs, such as the Chat Generative Pretrained Transformer (ChatGPT), which has the ability to read large volumes of literature and generate responses like an experienced reader. Despite the great potential of LLMs, their use in healthcare is not very widespread. This is mainly due to the risk of distortion, especially for references, which has been particularly recognised for ChatGPT4, but also for other LLMs.^17^

The ability of LLMs to perform a wide range of language tasks, such as answering questions, writing articles, translating languages, and building conversational agents, makes them particularly valuable tools for use in many scientific fields. LLMs are expected to be widely used in rheumatology in the future. This use is based on the processing and analysis capabilities of vast amounts of medical literature, allowing clinicians and researchers to stay up-to-date with the latest developments in rheumatology. In addition, they can help create personalised educational materials for patients, ensuring that patients receive accurate and actionable information about their treatments.^22^

## METHODOLOGY AND DATA

In this study, the methodology and data analysis were executed using the VOSviewer tool and the Python programming language. The detailed procedures for bibliometric analysis are elaborated in the following six subsections.

### Dataset

The survey covers the time frame from 1 January 2010 to 31 December 2024. The construction of the database was based on the use of Scopus keywords, and detailed information about the database is presented in **Table 1**. The decision to start the analysis in 2010 was motivated from the objective to capture a thorough and contemporary understanding of the subject.

**Table 1.** Data retrieval constraints and parameters for the Scopus database.

**Data**	**Search Criteria and Results**
Database	Scopus
Search field	Title, Abstract, Keywords
Keywords	Artificial Intelligence, machine learning (ML), Large Language Models (LLMs) rheumatology, rheumatoid arthritis, osteoarthritis, spondyloarthritis, rheumatic diseases
Years	2010–2024
Author name	All
Subject Area	All
Document type	Article
Source title	All
Affiliation	All
Funding sponsor	All
Country	All
Source type	Journal
Language	English
Publication stage	Final
Open Access	All open access
Scopus query	TITLE-ABS-KEY “artificial intelligence”, OR “machine learning” OR “large language models” OR “LLMs”, AND “rheumatology”, OR “rheumatoid”, OR “arthritis”, OR “osteoarthritis”, OR “spondyloarthritis”, OR “rheumatic”, AND “diseases”) AND PUBYEAR > 2009 AND PUBYEAR < 2025 AND ( LIMIT- TO ( DOCTYPE, “ar”)) AND ( LIMIT-TO ( SRCTYPE, “j” ) ) AND ( LIMIT TO ( LANGUAGE, “English” ) ) AND ( LIMIT-TO ( PUBSTAGE, “final” ) ) AND ( LIMIT-TO ( OA, “all” )
Data extracted	December 31, 2024
Number of publications	1268

This starting point was chosen because advances in technology affecting medicine have been particularly intense in recent years.^4^

An extensive search for English language articles focusing on Applications of AI in Rheumatology was performed using the Scopus database. Articles that were not in English language were excluded using filters in the Scopus database. The Scopus database was searched for articles that included at least one term from the group “artificial intelligence”, “machine learning”, “large language models” or “LLMs”, and at least one term from the group “rheumatology”, “rheumatoid”, “arthritis”, “osteoarthritis”, “spondyloarthritis”, “rheumatic”, along with the word “diseases”.

**Figure 1.**
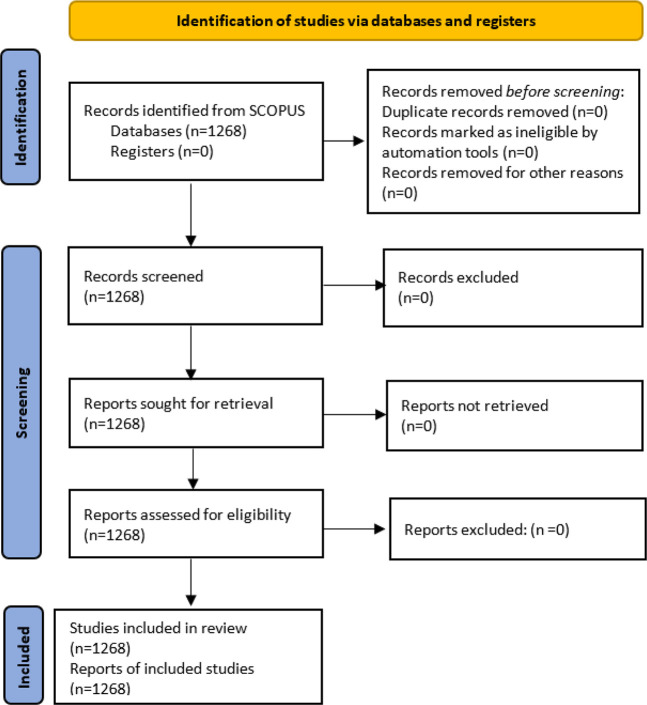
Flow chart of the study selection process.

This means that an article was only included if it mentioned both a concept from AI and a concept from rheumatology. The study selection process is illustrated in the flowchart (**Figure 1**), which was created according to the Preferred Reporting Items for Systematic Reviews and Meta-Analyses (PRISMA) guidelines.^18^

It is important to note that, unlike typical systematic reviews, this bibliometric analysis did not involve a step-by-step assessment of the full content of individual articles for inclusion. In contrast, the depicted flow reflects the data retrieval process from the Scopus database, where initial records (n=1268) were identified using a predefined query and then refined through the application of database filters (as detailed in **Table 1**). While the PRISMA flowchart provides a structured overview of data retrieval, its direct application is limited by the inherent limitations of bibliometric analyses, which focus on metadata rather than in-depth content assessment. Also, this bibliometric review was conducted and reported according to the BIBLIO checklist for bibliometric studies.^19^ The completed BIBLIO checklist is provided in the **Appendix**.

It is noted that ethical considerations were maintained throughout the study process, ensuring that copyright and citation rules were adhered to, while using data from the existing literature. After the initial search, the extracted data were exported to a text file for further analysis.

### Citation analysis

Bibliometric analyses are based on two major units: the scientific publication as an indicator of research output, and citations received by them as a proxy of their scientific impact or influence on the scholarly community. Citation analysis stands as a prevalent technique in bibliometric analysis. It involves evaluating the impact and influence of a specific paper or author in a particular field of study by quantifying the frequency with which the paper or author is referenced in other scholarly works.^18^

### Co-authorship analysis

In general, co-authorship is a key mechanism that links different sets of talent to produce a research output. It stands out as a widely acknowledged method for unveiling collaborative patterns among authors, institutions, and countries across various fields of study. It aids in identifying influential figures, institutions, and emerging trends within the subject of inquiry.^23,24^

### Bibliographic coupling analysis

In our case, bibliographic coupling analysis serves to explore the interconnectivity and collaborative synergy among diverse scientific publications, relying on the commonality of their cited references. The strength of their relatedness (coupling) becomes more when a document receives more citations. It provides the similarities of the two works’ subject matter in the form of documents, source, author, organisation, and countries.^23, 24, 25^

### Co-occurrence analysis

Co-occurrence analysis focuses on analysing counts of co-occurring entities within a collection of units. It is simply the counting of paired data within a collection unit, and proves to be a valuable method for discerning connections between keywords or concepts frequently found together in literature.^4,24^ The approach to author co-occurrence entails pinpointing the most commonly appearing keywords or concepts within a given set of papers.

### Co-citation analysis

Document co-citation is used to conduct searches on similar documents. Journal co-citation is of interest to the collection manager concerned with core journal lists, journal selection, and evaluation of collections serving specific research-oriented groups. Author co-citation has been used to analyse the intellectual structure of scientific disciplines.

## RESULTS

### Overview of the historical trends

**Figure 2.**
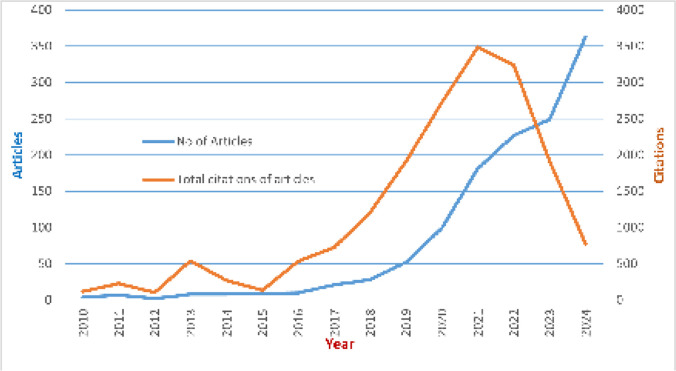
The number of publications and total citations from 2010 to 2024.

The historical trends of the number of annual published articles and the total frequency of citations about the use of artificial intelligence und rheumatology between 2010 and 2024 are shown in **Figure 2**. According to Figure 2, the number of annual publications has continued to increase over the past 14 years: in the early 2010s, the number of publications has remained relatively constant started to increase from the year 2017, followed by faster growth till 2024. The conclusion that emerges from a macroscopic view of **Figure 2** is that the interest in the use of AI in rheumatology shows a very large increase after 2017. This trend is considered logical and expected, given that developments in information technology and applications of AI in the healthcare sector have been rapid in this 6-year period. Whereas, the number of annual reports increased with some fluctuations during the period 2010 - 2017, followed by a sharp decrease after the year 2021. This is expected because there is a significant lag for academic publications to have a measurable impact, as it takes time for others to read new studies, notice and cite these new studies to publish their own work. Thus, articles published in more recent years naturally have a lower number of citations.

### Results of citation analysis

The results of the citation analysis about “AI and rheumatology” are presented in **Table 2**, which provides the structure of citations in the relevant research area by presenting the top 10 documents with the highest number of citations until 31 December 2024. The table highlights the authors, title, and the number of citations for each document. These documents represent significant contributions to the field of rheumatology, AI, ML, and other related topics. Topping the list is the paper by Forbes et al titled “A comparative study of the gut microbiota in immune-mediated inflammatory diseases - Does a common dysbiosis exist?” citation counts of 310. Then follow the articles Stebbing et al.,^27^ Huang et al.,^28^ Kishikawa et al.,29 Pierson et al.,30 each contributing significantly to the discourse with citation counts of 230, 192, 172, and 166, respectively.

**Table 2.** Results of the top 10 documents by citations (until 31 December 2024).

**No.**	**Reference**	**Title**	**Citations**
1	Forbes et al., 2018	A comparative study of the gut microbiota in immune-mediated inflammatory diseases - Does a common dysbiosis exist?	310
2	Stebbing et al., 2020	Mechanism of baricitinib supports artificial intelligence-predicted testing in COVID-19 patients	230
3	Huang et al., 2013	Autoimmune, atopic, and mental health comorbid conditions associated with alopecia areata in the United States	192
4	Kishikawa et al., 2020	Metagenome-wide association study of gut microbiome revealed novel aetiology of rheumatoid arthritis in the Japanese population	172
5	Pierson et al., 2021	An algorithmic approach to reducing unexplained pain disparities in underserved populations	166
6	Tiulpin et al., 2019	Multimodal Machine Learning-based Knee Osteoarthritis Progression Prediction from Plain Radiographs and Clinical Data	162
7	Norgeot et al., 2019	Assessment of a Deep Learning Model Based on Electronic Health Record Data to Forecast Clinical Outcomes in Patients With Rheumatoid Arthritis	160
8	Rivellese et al., 2022	Rituximab versus tocilizumab in rheumatoid arthritis: synovial biopsy-based biomarker analysis of the phase 4 R4RA randomised trial	145
9	Ornage et al., 2018	Identification of Three Rheumatoid Arthritis Disease Subtypes by Machine Learning Integration of Synovial Histologic Features and RNA Sequencing Data	145
10	Oakden-Rayner, 2019	Exploring Large-scale Public Medical Image Datasets	130

These papers span a range of topics that concerns the use of AI and ML to immune-mediated inflammatory diseases and rheumatology. The diversity of the top-cited documents underscores the multidimensional nature of research in the field, capturing key contributions that have garnered substantial attention within the scholarly community.

**Table 3** provides insights into the top 10 authors, countries, and journals by the number of publications until 31 December 2024. These tables provide a comprehensive overview of the most influential authors, and countries, in the field, offering valuable insights into the landscape of academic research and publication. In the table of authors, to exclude those who do not play a central role in the respective papers, only authors listed as the first author are presented. Leading the list of authors is Bonakdari Hossein with 5 publications. Following Hugle Thomas, Kim Woorim, Venerito Vincenzo, Queiro Ruben, and Lip Gregory with 4 publications. Shifting the focus to countries, United States emerges as the dominant force, occupying the top spot with a remarkable 309 publications. The China and United Kingdom follow with substantial contributions of 309 and 160 publications, respectively. Germany and India are included in the top-5, emphasising their active involvement in the global research landscape.

**Table 3.** Results of the top 10 authors and countries by publications (until 31 December 2024).

**No.**	**Author**	**Number of Documents**	**No.**	**Country**	**Number of Documents**
1	Bonakdari, Hossein	5	1	United States	394
2	Hügle, Thomas	4	2	China	309
3	Kim, Woorim	4	3	United Kingdom	160
4	Venerito, Vincenzo	4	4	Germany	97
5	Queiro, Rubén	4	5	India	75
6	Lip, Gregory Y. H.	4	6	Canada	74
7	Hammam, Nevin	3	7	Netherlands	72
8	Madrid-García, Alfredo	3	8	Spain	71
9	Morales-Ivorra, Isabel	3	9	France	64
10	Koo, Bon San	5	10	Italy	60

**Figure 3.**
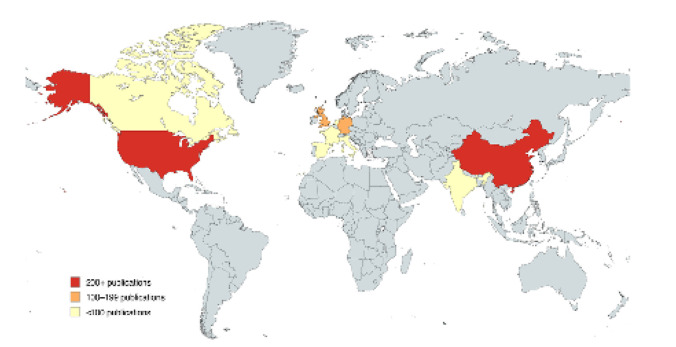
Geographic distribution of top 10 leading countries during 2010 and 2024.

For a better overview of and geographical distribution, **Figure 3** has been constructed, which depicts significant contributions of the top 10 countries in the literature related to AI and rheumatology, ranked by the number of publications related to the keywords used. It is noted that the national ownership of a work depends on the full address of its first author, when multiple authors come from different countries.

### Results of co-authorship analysis

**Figure 4.**
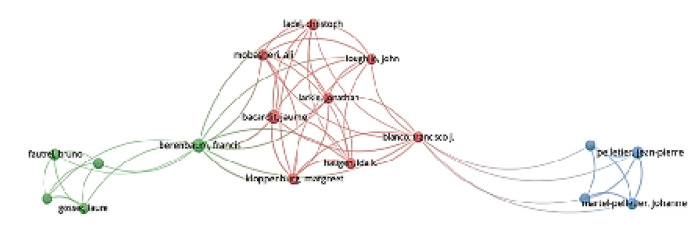
Co-authorship analysis on authors.

**Figure 5.**
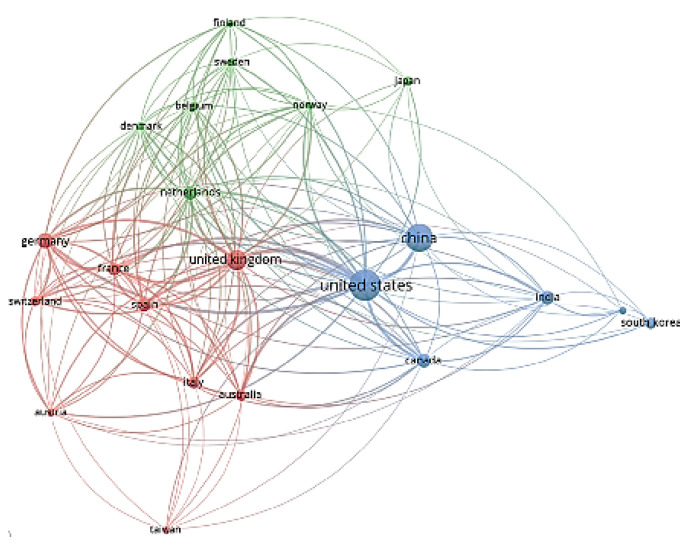
Co-authorship analysis on countries.

**Figure 4** presents an author co-authorship map derived from the analysed dataset, which represents the cooperative relationships between authors in the field of AI, ML, LLMs, rheumatology, and autoimmune diseases. The connections between points on the map represent the co-authorship between authors, while the distance between clusters indicates the strength of the co-authorship relationship. The author’s co-authorship map provides a visual representation of cooperative relationships, enabling a better understanding of the collaboration patterns and the strength of relationships. Out of the 8129 identified authors, 40 exceeded the established threshold of 5 co-authored works. The figure highlights the largest connected set, encompassing 17 items. The higher total link strengths are for Berenbaum (42), Bacardit (40), and Kloppenburg (35). There are three clusters: Cluster 1 includes Bacardit (40), Blanco (31), Haugen (27), Kloppenburg (35), Ladel (28), Larkin (32), Loughlin (27), Mobasheri (33). Cluster 2 includes Berenbaum (42), Fautrel (6), Gossec (11), Pandit (7), Servy (11). Cluster 3 includes Abram (15), Bonakardi (15), Martael-Pelletier (17), Pelletier (17). **Table 4** shows some of the data of co-authorship, specifically, the names of the authors, the number of documents, the number of citations and the number of total link strength. From the elements of **Table 4**, the creation of the 3 clusters described previously is evident. **Figure 5** presents a country co-authorship map derived from the analysed dataset, which represents the cooperative relationships between countries in the field of research. The connections between points on the map represent the co-authorship between countries, while the distance between clusters indicates the strength of the co-authorship relationship. The country authorship map provides a visual representation of cooperative relationships, enabling a better understanding of the collaboration patterns and the strength of relationships. Out of the 112 identified countries, 22 exceeded the established threshold of 20 documents.

**Table 4.** Results of co-authorship between authors.

**Clusters**	**Documents**	**Citations**	**Total link strength**
**Cluster 1 (8 items)**			
Bacardit, Jaume	8	206	40
Blanco, Francisco J.	5	65	31
Haugen, Ida K.	5	59	27
Kloppenburg, Matgreet	6	136	35
Ladel, Cristoph	5	139	28
Larkin, Jonathan	5	176	32
Loughlin, John	5	113	27
Mobasheri, Ali	6	195	33
**Cluster 2 (5 items)**			
Berenbaum, Francis	8	224	42
Fautrel, Bruno	5	161	6
Gossec, Laure	5	238	11
Pandit, Aridaman	5	209	7
Servy, Herve	5	238	11
**Cluster 3 (4 items)**			
Abram, Francois	5	99	15
Bonakardi, Hossein	5	83	15
Martael-Pelletier, Johanne	6	111	17
Pelletier, Jean-Pierre	6	111	17

In detail, the four clusters created and their data are shown in **Table 5**. As shown in **Figure 5** and **Table 5**, the largest cluster, represented in red (cluster 1), consists of 9 countries, followed by the green cluster (cluster 2) with 7 items, the blue cluster (cluster 3) with 6 countries. It is crucial to search deeper into the clusters identified, as they offer valuable insights into the collaborative networks among countries.^20,25^

**Table 5.** Results of co-authorship between countries.

**Clusters**	**Documents**	**Citations**	**Total link strength**
**Cluster 1 (9 items)**			
Australia	48	874	108
Austria	20	313	67
France	64	1188	176
Germany	97	1484	206
Italy	60	1215	125
Spain	71	1141	173
Switzerland	44	484	96
Taiwan	24	250	15
United Kingdom	160	3290	301
**Cluster 2 (7 items)**			
Belgium	25	515	86
Denmark	28	572	69
Finland	20	497	60
Japan	34	477	15
Netherlands	72	1504	181
Norway	20	373	83
Sweden	25	669	61
**Cluster 3 (6 items)**			
Canada	74	1549	105
China	309	2736	109
India	75	935	52
Saudi Arabia	20	133	15
South Korea	47	596	11
United States	394	7782	376

The higher total link strengths are for United States (376), United Kingdom (301), Germany (206), Netherlands (181) and France (176). The total link strengths, as indicated by the relevant numbers, reflect the cumulative collaboration strength between countries in the co-authorship network. Higher values signify a greater volume of collaborative research output. Notably, countries like the European countries United Kingdom, Germany, the Netherlands, and France, as well as the United States, exhibit exceptionally high total link strengths, indicating prolific collaboration and substantial contributions to global research networks in the field. These countries, along with others listed, emerge as key contributors with extensive international research partnerships, underscoring their prominent roles in the collaborative landscape of the examined research domain.

**Figure 6.**
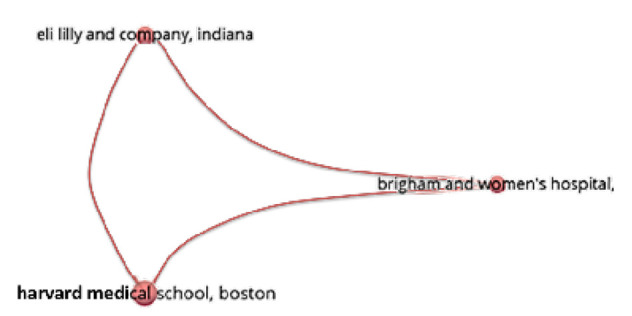
Co-authorship analysis on organisations.

In conclusion, the co-authorship analysis reveals distinct collaborative patterns among countries in the field of AI, ML, rheumatology, and rheumatologic diseases. The variations among clusters underscore the dynamic and interconnected nature of international collaborations in advancing research within the specified field. **Figure 6** illustrates the results of co-authorship analysis, focusing on Organisations. Out of the 5660 identified countries, 10 exceeded the established threshold of 4 documents. **Figure 6** and **Table 6** both highlight the largest connected set, consisting of 9 items, instead of displaying all the items. The cluster consists of organisations that belong to USA.

**Table 6.** Total link strength of co-authorship between organisations.

**Clusters**	**Documents**	**Citations**	**Total link strength**
**Cluster 1 (3 items)**			
Brigham and Women’s Hospital, Boston, USA	4	49	2
Eli Lily and Company, Indianapolis, USA	5	278	3
Harvard Medical school, Boston, USA	9	273	3

### Results of bibliographic coupling analysis

**Figure 7.**
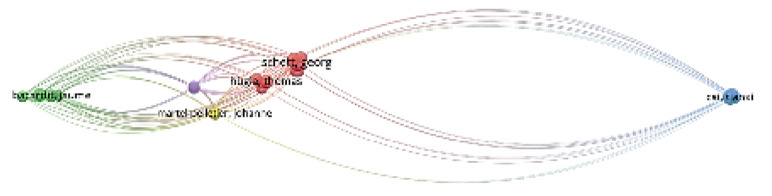
Bibliographic coupling analysis of the influential authors’ total link strength.

**Figure 7** provides a detailed overview of the bibliographic coupling network analysis, revealing intricate connections among authors within the Scopus dataset.

With a minimum threshold of 6 citations per document, 19 authors out of the 8129 identified met the specified criteria. A significant outcome of the bibliographic coupling analysis is the identification of authors with the highest total link strengths in the network, as arises from **Table 7**. Noteworthy contributors include Bacardit J. (1092) with 8 articles, Mobasheri A. (906) with 6 articles, and Berenbaum F. (905) with 8 articles. These authors play pivotal roles within the network, signifying their substantial contributions and collaborations in the field of AI, ML, rheumatology, and rheumatologic diseases.

**Table 7.** Results of the influential authors and total link strength from the bibliographic coupling network analysis.

**Clusters**	**Documents**	**Citations**	**Total link strength**
**Cluster 1 (8 items)**			
Davis, John M.	6	136	97
Hugle, Thomas	8	44	69
Kleyer, Arnd	8	142	749
Knitza, Johannes	10	149	621
Li, Jian	6	13	130
Schett, Georg	12	170	806
Venerito, Vincenzo	6	41	79
**Cluster 2 (4 items)**			
Bacardit, Jaume	8	206	1092
Berenbaum, Francis	8	224	905
Kloppenburg, Matgreet	6	136	890
Mobasheri, Ali	6	195	906
**Cluster 3 (3 items)**			
Cai, Tianrum	8	155	580
Cai, Tianxi	10	198	596
Hong, Chuan	6	99	501
**Cluster 4 (2 items)**			
Martael-Pelletier, Johanne	6	111	476
Pelletier, Jean-Pierre	6	111	476
**Cluster 5 (2 items)**			
Moustakidis, Serafeim	6	126	544
Tsaopoulos, Dimitrios	6	126	544

**Figure 8.**
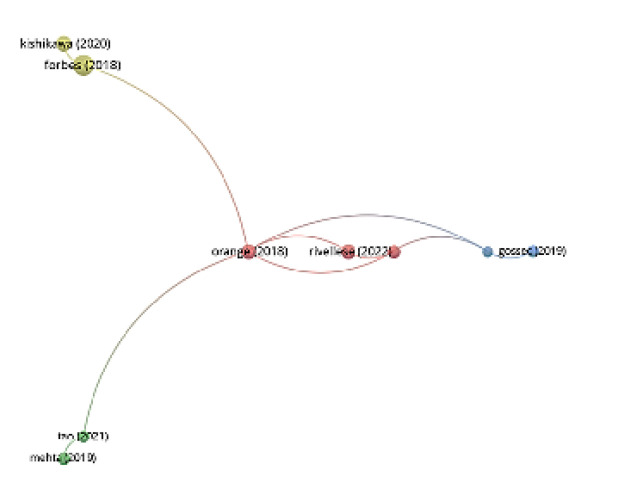
Bibliographic coupling analysis and the influential documents and total link strength.

The results of the bibliographic coupling analysis, focusing on documents with a minimum of 80 citations, reveal insightful clusters that underscore the interconnectedness of influential works within this research domain. Out of 1268 documents analysed, 25 met the defined thresholds, forming cohesive clusters of intellectual synergy. **Figure 8** highlights the largest connected set, consisting of 9 items, instead of displaying all the items. Additionally, **Table 8** includes the clusters, the citations and total link strength of each author.

**Table 8.** Results of the influential authors and total link strength from the bibliographic coupling network analysis.

**Clusters**	**Citations**	**Total link strength**
**Cluster 1 (4 items)**		
Orange (2018)	145	5
Rivellese (2022)	145	2
Yeo (2016)	114	3
**Cluster 2 (2 items)**		
Mehta (2019)	113	1
Tao (2021)	103	2
**Cluster 3 (2 items)**		
Gossec (2029)	105	1
Guan (2019)	95	3
**Cluster 4 (2 items)**		
Forbes (2018)	310	2
Kishikawa (2020)	172	1

### Results of Co-citation Analysis

**Figure 9.**
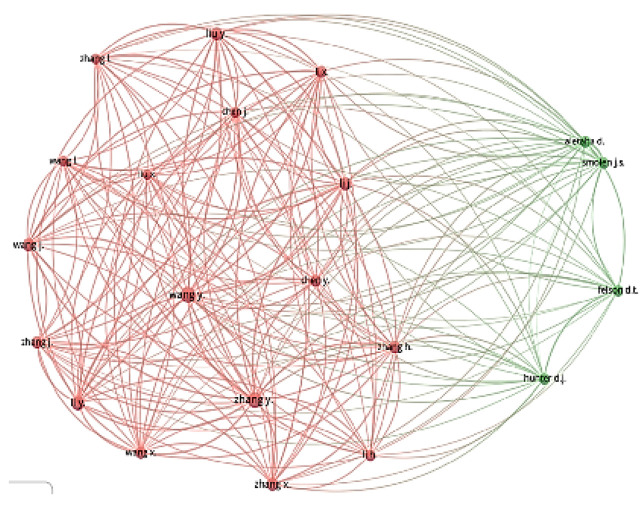
Co-citation analysis of influential authors.

**Figure 9** displays the results of the co-citation Analysis, with the unit of analysis set to cited authors. With a minimum threshold of 150 citations per author, 21 authors out of the 128936 authors identified met the specified criteria. The figure highlights the connected set. Notably, the authors with the highest total link strengths in the network are Wang, Y. (3182), and Zhang Y. (3054), as shown in **Table 9**. As depicted in **Figure 9**, the co-citation network is organised into three distinct clusters. Cluster 1 represented in red, consists of 17 authors, followed by the green Cluster 2 with 4 authors. **Table 9** provides an in-depth identification of collaborators within each cluster, along with their respective total link strengths obtained from the co-citation analysis. The identified clusters represent significant collaborative networks among authors, providing insights into the patterns of co-citation and the strength of relationships within the research community.

**Table 9.** Results of co-citation analysis of influential authors.

**Clusters**	**Citations**	**Total link strength**
**Cluster 1 (17 items)**		
Chen J.	168	1698
Chen Y.	194	1689
Li H.	168	1521
Li J.	234	2535
Li X.	208	2264
Li Y.	291	2707
Liu X.	160	1724
Liu Y.	226	2176
Wang J.	229	2304
Wang L.	161	1597
Wang X.	205	2063
Wang Y.	334	3182
Zhang H.	207	1946
Zhang J.	200	1964
Zhang L.	160	1780
Zhang X.	224	1962
Zhang Y.	347	3054
**Cluster 2 (4 items)**		
Aletaha D.	193	1127
Felson D.T.	171	838
Hunter D.J.	176	824
Smolen J.S.	182	1043

**Figure 9** shows a high degree of co-citation among Asian authors, which may be due to the high degree of collaboration among these authors, as well as the large number of relevant journals that have appeared in recent years in Asian countries. The strong representation of Asian authors may also be attributed to other factors, such as the large population of Asian countries, the increasing research output from institutions in countries such as China, and the significant investments in artificial intelligence and biomedical research.

### Results of Co-occurrence analysis

**Figure 10.**
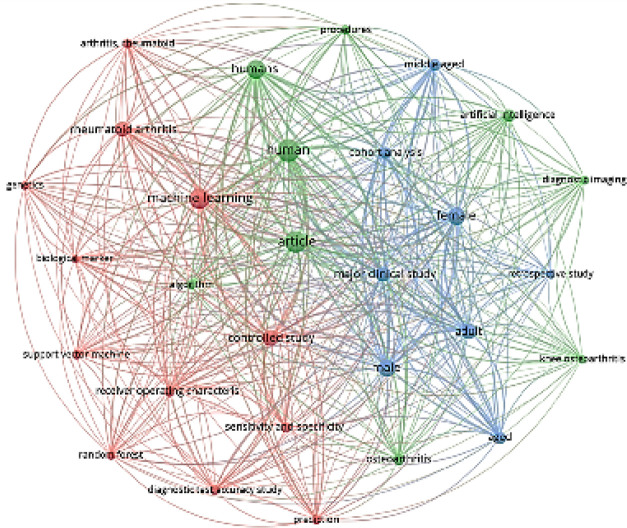
Co-occurrence analysis with a minimum threshold of 100 occurrences.

A comprehensive co-occurrence analysis with a minimum threshold of 150 occurrences for keywords reveals intricate patterns and associations among key terms and 4 distinctive clusters as displayed in **Figure 10**. Of the 13091 keywords, 29 meet the threshold. Notably, the keywords with the highest link strengths include “Human”; “Article”; “Machine learning”; “Humans”; “Female”; “Male”; “Adult”; “Controlled study”; “Major clinical study” and “Rheumatoid arthritis”, underlining their centrality in the scholarly discourse.

The co-occurrence analysis identifies four distinct clusters, each representing a thematic concentration within the field. Cluster 1 (in red) focuses on rheumatoid arthritis, machine learning, and diagnostic methods. Includes terms related to disease diagnosis (sensitivity and specificity, receiver operating characteristic) and machine learning techniques (random forest, support vector machine), highlighting the role of artificial intelligence in medical research. Cluster 2 (in green) is centred on artificial intelligence applications in medicine. Key terms include algorithm, artificial intelligence, and procedures, alongside general biomedical concepts like human, knee osteoarthritis, and osteoarthritis, indicating its relevance to broader medical research. Cluster 3 (8 items) is related to demographics and clinical studies, featuring terms such as adult, aged, female, male, and middle aged. It also includes research methodologies like cohort analysis and retrospective study, emphasising population-based studies in medical research. The above results are presented in detail in **Table 10**. The absence of prominent imaging-related terms (e.g., magnetic resonance imaging, ultrasonography, radiography) from Cluster 1 (red, rheumatoid arthritis-focused) and their relative association with Cluster 2 (green, broader “artificial intelligence in medicine”) reflects important underlying patterns in the current literature.

**Table 10.** Results of co-occurrence analysis.

**Clusters**	**Occurrences**	**Total link strength**
**Cluster 1 (12 items)**		
Arthritis, rheumatoid	196	2067
Biological marker	186	1001
Controlled study	577	6256
Diagnostic test accuracy study	205	2476
Genetics	168	1618
Machine learning	895	7991
Prediction	196	2111
Random forest	181	2014
Receiver operating characteristic	254	2971
Rheumatoid arthritis	469	4170
Sensitivity and specificity	235	2760
Support vector machine	187	1963
**Cluster 2 (9 items)**		
Algorithm	220	2264
Article	1023	9597
Artificial intelligence	322	2400
Human	1075	9924
Humans	791	7587
Knee osteoarthritis	178	1642
Osteoarthritis	296	2619
Procedures	194	1944
**Cluster 3 (8 items)**		
Adult	552	6320
Aged	321	3778
Cohort analysis	268	3175
Female	649	7251
Major clinical study	492	5706
Male	646	7165
Middle aged	327	3945
Retrospective study	159	1859

In conclusion, the co-occurrence analysis offers valuable insights into the thematic concentrations and interconnections within the diverse landscape of rheumatologic studies. It reveals a nuanced research domain, characterised by the identification of four distinct clusters. These clusters underscore the multidimensional nature of rheumatology, rheumatologic diseases, AI, Learning Machine, Osteoarthritis, Rheumatoid Arthritis etc.

## STRENGTHS AND LIMITATIONS

This study includes a bibliometric analysis focusing on the application of AI in rheumatology, providing a guide for researchers in related research areas. The research methods used included the application of several commonly used bibliometric techniques, such as citation analysis, co-authorship analysis, co-occurrence analysis, and bibliographic linkage analysis. Also, by using VOSviewer, which is a widespread tool in bibliometrics, the objectivity of the data analysis is ensured, while the analysis provided deeper insights into the research centres and the main points of the relationship between AI and rheumatology.

We consider it necessary to recognise the following inherent limitations of the bibliometric approach: (a) The data search was performed using the Scopus database, which is the most important and largest database of interdisciplinary academic literature worldwide. The decision to use Scopus as the source for this study was based on its extensive indexing of high-quality, peer-reviewed journals and its robust metadata, which includes abstracts, citations, and keywords. It is possible that there are other relevant publications in other smaller databases. The scope of findings could be expanded by incorporating other established databases, such as the Web of Science (WoS) database. (b) The use of keywords can be puzzling. (c) Potential limitations arise from the exclusion of non-English language articles, since the search was performed in studies written in English, which results in the non-inclusion of some important publications in another language. Finally, the study database was limited to: i) articles as document type and ii) journals as source type. c) The findings regarding collaborations include only the name of the first author, while the country collaboration networks are not analysed.

## DISCUSSION - CONCLUSIONS

The scientific literature in the field of AI and its specific subset techniques has grown rapidly in the last decade, due to the exponential increase in computing power and data storage capacity. The research launched an exploration of the landscape around AI and rheumatology. The citation analysis revealed a wide range of highly cited papers covering topics related to the use of AI and Machine Learning in immune-mediated inflammatory diseases, rheumatoid arthritis and musculoskeletal diseases. This diversity underscores the multidimensional nature of research in the field, eliciting important contributions that have garnered substantial attention in the scientific community.

The co-authorship analysis illuminated complex networks of collaboration between countries. Emerging groups such as the European cluster and the USA cluster highlighted the dynamic and interconnected nature of international collaborations, offering valuable insights into the global landscape of research in this scientific field. The analysis of co-authorship, focusing on the Organisations showed the clusters of countries to which the examined Organisations belong. In this case, Organisations belonging to European countries appear to dominate. Co-occurrence analysis identified four thematic clusters, emphasising the intersection of AI, ML, rheumatology, and rheumatic diseases in general. These groups provided an understanding of the multifaceted nature of studies in the scientific field under consideration.

It is noted that the use of keywords shapes the results of the research, while different keywords could yield different results. Additionally, restriction to a particular database introduces bias. This study serves as a directional guide rather than an exhaustive exploration, recognising the evolving nature of research and the emergence of new terminologies. While this study provides a basis for understanding the current state of research on AI, ML and rheumatology, there is a need for further refinement and improvement. As the field evolves, continuing to adapt research methodologies and explore emerging issues will be critical to advancing the scientific discourse and addressing the complex challenges in this field.

By attempting a comparison of the content of the analysis and the results of this study and three other related studies conducted in the last two years, we conclude the following:

Liu et al. (2024)^10^ limited themselves to the literature concerning the application of TA in Rheumatoid Arthritis. The main conclusions that coincide between the study of Liu et al. (2024) and the present study concern the large increase observed in relevant publications in recent years worldwide, as well as the leadership of the USA and China in terms of the number of publications. Similar are the results of the study of Zhao et al. (2024),^11^ who investigated the application of TA in rheumatic diseases. In this analysis too, the number of relevant publications shows an increasing trend in recent years, while the USA leads in the number of publications compared to other countries. Finally, the content of the analysis by Polyzou and Baraliakos (2025)^12^ differs from the content of the present analysis and the aforementioned articles, as it concerns the calculation of different bibliometric indicators of the quantitative evolution of the literature dealing with the application of AI in rheumatology. This article also finds that the use of Artificial Intelligence (AI) in rheumatology has shown increasing trends in the last 5 years, according to the number of relevant articles published in scientific journals during the period 2010–2024. This trend is considered logical and expected, given that developments in information technology and AI applications were rapid in this 5-year period.

We believe that the findings of the article will have a significant contribution to the promotion of research and the general reflection in the Mediterranean rheumatology community regarding the management of rheumatic diseases in Mediterranean countries. It is worth mentioning that Mediterranean countries are characterised by a high incidence of rheumatic diseases, in which genetic, environmental and phenotypic heterogeneity pose significant diagnostic and therapeutic challenges.^31–34^ Although the contribution of Mediterranean countries to the global volume of publications in AI and rheumatology research compared to North American and North European groups is not particularly large, the emerging networks of co-authors connecting these countries with larger European and North American hubs signal increasing integration into international collaborative frameworks.

In conclusion, the bibliometric analysis performed in this article illustrates research trends related to the use of AI in rheumatology, providing information on the evolution of academic knowledge. It can be used as a “tool” by researchers, institutions and policymakers to make informed decisions, identifying emerging topics, gaps in the literature and evolving practices. The results of the calculations allow researchers to map co-authorship networks, co-citation patterns, and keyword co-occurrence, helping to visualise the connections between different research fields, different countries, and collaborations.

As mentioned above, the data for the article analysis were obtained using the Scopus database, which, while comprehensive and widely considered a powerful source for bibliometric analyses in the health sciences, future research using other databases, such as PubMed/MEDLINE, Web of Science, and Dimensions, may provide a more comprehensive and balanced picture of global research output and collaborative networks in this rapidly evolving field.

## Data Availability

The datasets used and analysed during this study are available from the corresponding author upon reasonable request.

## APPENDIX

https://mjrheum.org/assets/files/supplementary/MJR-2025-0366_APPENDIX.pdf
